# Attitudes, usage patterns, and learning interests of medical students toward DeepSeek in medical education: A cross-sectional survey

**DOI:** 10.1097/MD.0000000000049785

**Published:** 2026-07-17

**Authors:** Jing Ni, Fan Yang, Wenjie Xuan, Zhuoyi Wu, Lingqiong Jiang

**Affiliations:** a Department of Epidemiology and Biostatistics, School of Public Health, Anhui Medical University, Hefei, Anhui, China; b Department of Pathophysiology, School of Basic Medical Science, Anhui Medical University, Hefei, Anhui, China; c Experimental Teaching Center, Anhui Medical University, Hefei, Anhui, China.

**Keywords:** artificial intelligence, attitudes, Deepseek, learning interests, medical education, usage patterns

## Abstract

The integration of artificial intelligence (AI) tools like DeepSeek into scientific research offers new opportunities for efficiency and innovation. However, attitudes and adoption among medical students remain underexplored. This study aims to investigate medical students’ attitudes, usage patterns, and interest in learning with DeepSeek. A cross-sectional online survey was conducted among medical students from various academic levels and fields. The questionnaire assessed demographics, attitudes, usage frequency and purposes, and learning interests. Data were analyzed using descriptive statistics and independent sample t-tests. Among 589 respondents, most were familiar with DeepSeek (86.08%), and 70.29% used it. A majority held positive attitudes toward DeepSeek, agreeing that it is useful for academic success (77.91%), makes research easier (78.37%), and will play an important future role (74.51%). However, concerns included reliability (61.34%), overreliance (48.51%), and privacy risks (51.88%). Usage varied: 28.23% used DeepSeek frequently, while 29.76% had never used it. Common uses included problem-solving (406 users) and literature search (256 users). Over 80% expressed interest in learning to use DeepSeek more effectively. No significant differences in attitudes were found across demographic groups. Medical students view DeepSeek as a promising tool for academic and research support but have significant concerns regarding reliability and ethical use. There is strong interest in structured learning opportunities. Integrating AI literacy into medical education and providing targeted training are recommended to promote responsible and effective use.

## 1. Introduction

The integration of artificial intelligence (AI) tools in scientific research has become increasingly prevalent, offering new opportunities for efficiency and innovation.^[1-3]^ Founded in January 2025, DeepSeek, unlike many commercial AI tools (e.g., ChatGPT), is completely free and open-source, which lowers the barrier to entry for people, especially those in resource-limited settings. Second, its training corpus includes a substantial proportion of Chinese medical literature and clinical cases, potentially offering greater relevance to the local medical education context. As a result, DeepSeek has become a sensation, capable of assisting researchers with various tasks such as data analysis, literature review, and problem-solving, thus attracting widespread attention.^[4,5]^ Since its initial launch, DeepSeek has been fast and easy to use, quickly becoming an invaluable tool for medical faculty, patients, students, and healthcare providers.^[6,7]^

Medical students, particularly those at different stages of their academic careers (e.g., undergraduates vs postgraduates), may have varying levels of familiarity and comfort with AI tools. While some students may embrace DeepSeek as a valuable resource for enhancing their research capabilities, others may harbor concerns about its reliability, ethical implications,^[8]^ or potential to undermine critical thinking skills.^[9]^ Additionally, factors such as academic background, research experience, and prior exposure to similar technologies (e.g., ChatGPT) may influence how students perceive and utilize DeepSeek.^[10]^ However, as an emerging practical tool, public awareness of DeepSeek remains relatively low, and there is currently a lack of effective empirical research to evaluate its prospects and practical value in medical education.

As artificial intelligence becomes increasingly integrated into medical education, it becomes ever more important to understand medical students’ attitudes, usage patterns, and learning interests regarding such technologies. To this end, from the perspective of medical students themselves, this study investigates the attitudes, usage patterns, and learning interests of undergraduate students in using DeepSeek, aiming to provide a reference for educators and policymakers to design and implement effective teaching interventions that meet the individual needs of students.

## 2. Materials and methods

### 2.1. Study design and participants

A cross-sectional survey of undergraduate medical students at Anhui Medical University was conducted using non-probability convenience sampling, and survey data collection occurred from March 2025 to May 2025. All undergraduates in the second semester of the 2024 to 2025 academic year who were willing to participate in the study were included. Those undergraduate students who refused to participate and those whose responses were incomplete (with more than 20% of the items missing) were excluded from the study. Ultimately, this survey included 589 undergraduate students from various majors such as basic medical science, clinical medicine, and preventive medicine.

### 2.2. Data collection

A self-designed questionnaire was used to assess the application of DeepSeek among undergraduate medical students in this study. The questionnaires were anonymously completed in the classroom. The questionnaire, developed by referencing previous literature, mainly consisted of 5 sections:

Demographic information: Includes questions on gender, age, research field, academic year, and publication history.

Attitudes and perceptions of DeepSeek: It consists of 11 questions and is designed with a 5-point Likert scale, ranging from 5 (strongly agree) to 1 (strongly disagree). The covered topics include assessing students’ familiarity with DeepSeek and their level of agreement with statements regarding its usefulness, reliability, and potential risks.

Usage patterns: Explores how frequently students use DeepSeek, the purposes for which they use it (e.g., problem-solving, data analysis, and literature review), and the specific features they utilize.

Learning interest: Understand students’ interest in learning with DeepSeek and figure out through which method they would prefer to learn and use DeepSeek in a more scientific way.

### 2.3. Ethical considerations

Participation in the survey is voluntary, and informed consent will be obtained from all participants. The study has been approved by the relevant institutional review board to ensure ethical compliance. By analysing the collected data, this study aims to provide a comprehensive understanding of how DeepSeek is perceived and utilized by medical students, as well as its potential to enhance scientific research in the medical field.

### 2.4. Data analysis

Mean and standard deviation (SD) were used to summarize the quantitative data, and frequencies were used to summarize the qualitative data. Attitude scores for DeepSeek (5-point scale) were summarized as mean ± SD and compared across demographics using an independent sample *T* test. The data were entered into Microsoft Office Excel for Windows, 2016. Data were then double-checked and transferred to the Statistical Package for Social Sciences, version 27 (IBM SPSS Statistics 27). Graphs were generated using Microsoft Office Excel. All statistical analyses were performed using 2-tailed tests and an alpha error of 0.05. A *P* value ≤ .05 was considered statistically significant.

## 3. Results

### 3.1. Basic characteristics

During the survey period, a total of 589 medical students answered the questionnaire. Most respondents (507, 86.08%) had heard of DeepSeek prior to the study, and 414 (70.29%) had used it. The mean age of the students was 21 years old. Based on this, age was categorized into 2 groups: below the mean and above the mean. The sample was relatively balanced in terms of gender, with 279 males (47.37%) and 310 females (52.63%). The majority of participants (457, 77.85%) were in clinical medicine, with a small proportion (130, 22.01%) in public health and management. Regarding grade level, 457 students (77.99%) were juniors and younger, and only a few (39, 6.62%) had published articles. The demographic information of the respondents is presented in Table 1.

**Table 1:** Basic characteristics data for the study participants.

Characteristics	N	%
Age (N = 589)		
<21 yr	236	40.07%
≥21 yr	353	59.93%
Gender (N = 589)		
Female	279	47.37%
Male	310	52.63%
Major (N = 587)		
Clinical medicine	457	77.85%
Public health & management	130	22.15%
Year of medical training (N = 586)		
≤3 yr	457	77.99%
≥5 yr	129	22.01%
Publication of papers (N = 589)		
Published articles	39	6.62%
Not yet published	550	93.38%
Have you heard of Deepseek before this study? (N = 589)		
Yes	507	86.08%
No	82	13.92%
Have you used of Deepseek before this study? (N = 507)		
Yes	414	70.29%
No	175	29.71%

### 3.2. Attitude towards the use of DeepSeek

**Figure 1.**
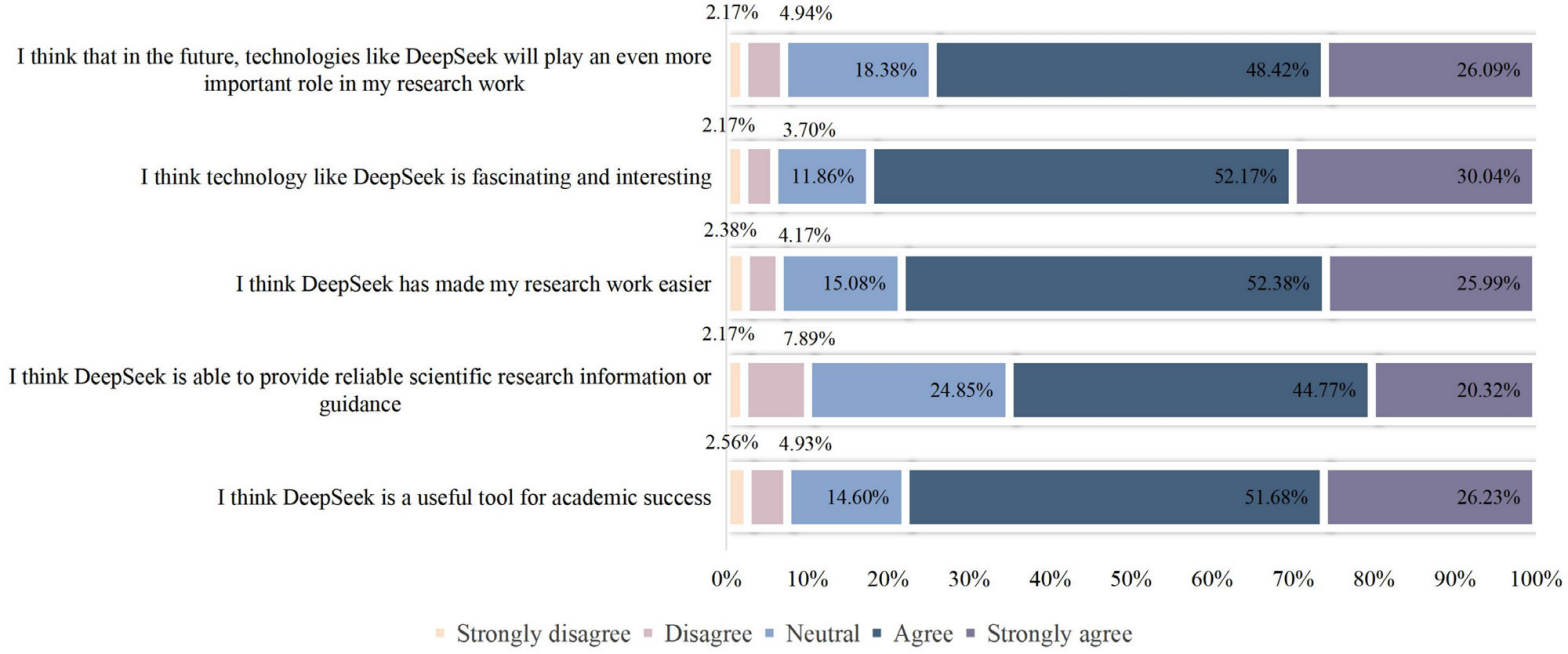
Positive attitudes towards DeepSeek among the study participants.

As shown in Figure 1, when evaluating medical students’ positive attitudes towards DeepSeek, the majority of students agreed or strongly agreed that “DeepSeek is a useful tool for academic success” (n = 77.91%). This was followed by “DeepSeek can provide reliable scientific research information or guidance” (n = 65.09%), “DeepSeek makes research work easier” (n = 78.37%), “Such technologies like DeepSeek are very attractive and interesting” (n = 82.21%), and “In the future, technologies like DeepSeek will play a more important role in research work” (n = 74.51%).

**Figure 2.**
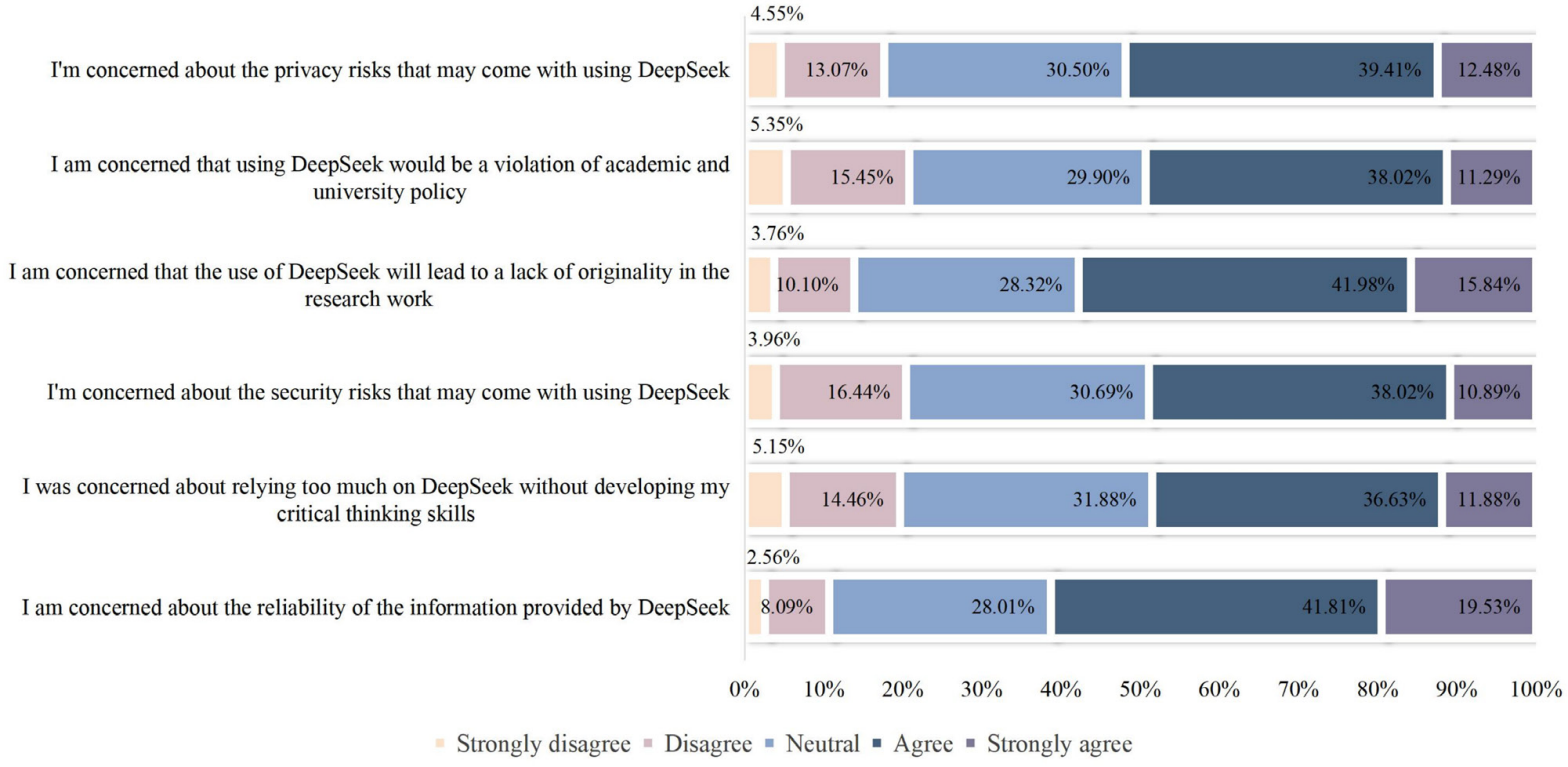
Negative attitudes towards DeepSeek among the study participants.

On the other hand, the participants’ views on the concerns regarding the use of DeepSeek indicate that students mainly focus on its accuracy and security. As a result, 311 students agreed or strongly agreed with “worrying about the reliability of the information provided by DeepSeek” (61.34%), and 245 students expressed “worrying about relying too much on DeepSeek without developing critical thinking skills” (48.51%). A total of 247 students agreed or strongly agreed with “the potential safety risks brought by using DeepSeek” (48.91%), 292 students were concerned that using DeepSeek would lead to a lack of originality in research work (57.82%); 249 students were concerned that “using DeepSeek would violate academic and university policies” (49.30%), and 262 students were concerned about “the privacy risks brought by using DeepSeek” (51.88%; Fig. 2).

### 3.3. Comparison of attitude scores in terms of basic characteristics

As shown in Table 2, although differences in positive and negative attitude scores toward DeepSeek were observed across groups based on age, gender, major, grade level, and publication history, none of these differences were statistically significant.

**Table 2:** Comparison of attitude scores in terms of basic characteristics.

Category		Group	Mean ± SD	*P* value
Positive attitude score	Age			.15
		≤20 yr	3.85 ± 0.96	
		>20 yr	3.95 ± 0.72	
	Gender			.67
		Female	3.93 ± 0.69	
		Male	3.90 ± 1.00	
	Major			.72
		Clinical medicine	3.91 ± 0.85	
		Public health & management	3.94 ± 0.78	
	Year of medical training			.72
		≤3 yr	3.91 ± 0.85	
		≥5 yr	3.94 ± 0.79	
	Publication of papers			.07
		Published articles	3.67 ± 1.52	
		Not yet published	3.93 ± 0.78	
Negative attitude score	Age			.65
		≤20 yr	3.43 ± 1.15	
		>20 yr	3.47 ± 0.96	
	Gender			.07
		Female	3.53 ± 0.89	
		Male	3.37 ± 1.19	
	Major			.05
		Clinical medicine	3.43 ± 1.05	
		Public health & management	3.52 ± 0.98	
	Year of medical training			.05
		≤3 yr	3.43 ± 1.06	
		≥5 yr	3.54 ± 0.95	
	Publication of papers			.26
		Published articles	3.29 ± 1.59	
		Not yet published	3.46 ± 0.99	

SD = standard deviation.

### 3.4. Practices of students in using DeepSeek

**Figure 3.**
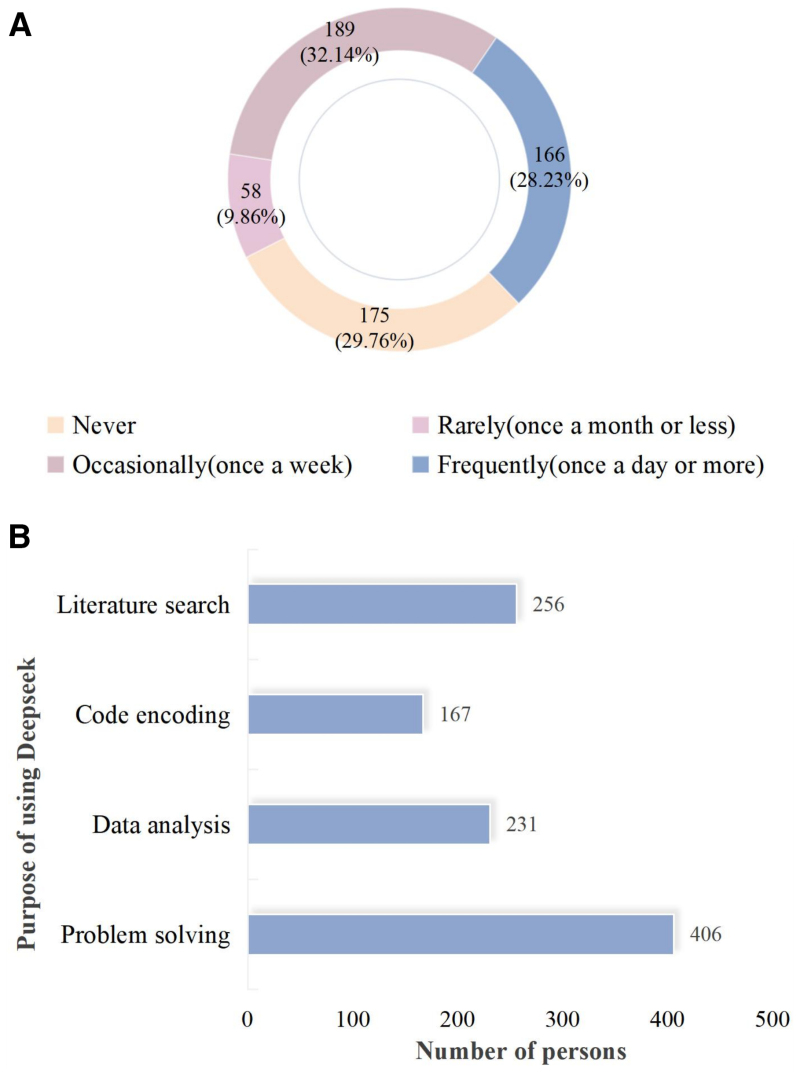
The frequency and type of using DeepSeek among the study participants. (A) The frequency of using DeepSeek among the study participants; (B) The type of using DeepSeek among the study participants).

The frequency of using DeepSeek varied among students (Fig. 3B). 28.23% of the students used it frequently (once a day or more). On the other hand, 29.76% of the participants had never used it (Fig. 3A). To date, 406 students have used DeepSeek to solve problems, 256 have used it to search literature, and 231 and 167 have used it to analyze data and write code, respectively.

### 3.5. Students’ interest in learning from DeepSeek

**Figure 4.**
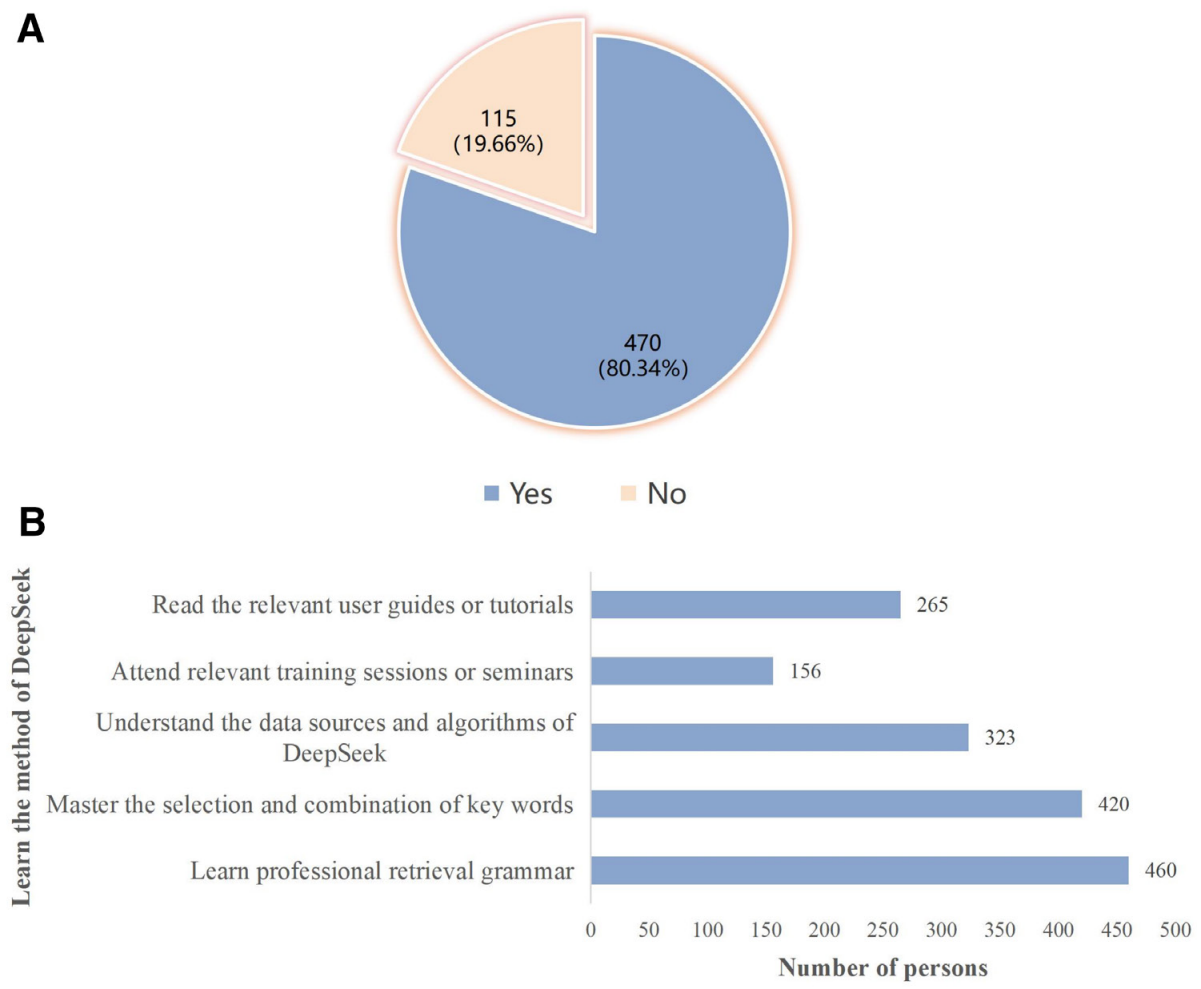
The willingness and methods of the study participants to learn Deepseek. (A) A study of the willingness of the participants to learn Deepseek; (B) A study of the willingness of the participants to learn the methods of Deepseek.

The majority (80.34%) of medical students expressed interest in learning from DeepSeek, demonstrating strong motivation for AI skill acquisition (Fig. 4A). Regarding the specific methods through which participants preferred to learn DeepSeek (Fig. 4B), “learning professional search grammar” is the most popular method, 460 students expressed interest in it. 420 students preferred “mastering the selection and combination of keywords”, and 326 students chose “understanding the data sources and algorithms of DeepSeek”. Meanwhile, 265 students were interested in “participating in relevant training courses or seminars”. While 256 students chose “reading relevant user guides or tutorials”.

## 4. Discussion

This cross-sectional survey provides valuable insights into attitudes, usage patterns, and learning interests related to DeepSeek among medical students. The findings highlight both the potential of DeepSeek as a supportive tool in academic and research settings, as well as the concerns that may hinder its widespread adoption.

The majority of students recognized DeepSeek as a useful tool for academic success, with over three-quarters agreeing that it facilitates research work and is both attractive and interesting. This positive reception suggests a readiness among medical students to integrate AI tools into their learning and research processes.^[11]^ The high percentage of students who believe that such technologies will play an increasingly important role in future research further underscores this openness.^[12]^

However, significant concerns remain, particularly regarding the reliability of the information provided by DeepSeek, potential safety risks, and the possibility of overreliance hindering the development of critical thinking skills. These concerns are consistent with broader ethical and practical debates surrounding the use of AI in academia.^[13,14]^ The fact that nearly half of the respondents expressed worries about originality, policy violations, and privacy risks indicates a need for clear guidelines and educational interventions to address these issues.^[15]^

Notably, no statistically significant differences in attitude scores were observed across demographic subgroups, including age, gender, major, grade level, or publication history. This suggests that perceptions of DeepSeek are relatively consistent across different segments of the medical student population, though the small number of students with publication experience may have limited the power to detect differences in this subgroup.

In terms of usage, a substantial proportion of students reported frequent use of DeepSeek, particularly for problem-solving and literature searches, while a notable minority had never used it. This variation in adoption may reflect differences in access, awareness, or comfort with AI technologies. The high percentage of students who had previously heard of or used ChatGPT suggests a general familiarity with conversational AI, which may facilitate the uptake of similar tools like DeepSeek.^[16]^

Importantly, students expressed strong interest in learning how to use DeepSeek more effectively and scientifically. This presents an opportunity for educators and institutions to develop targeted training programs – such as online courses, workshops, or seminars – that can help students leverage AI tools responsibly and efficiently.^[17,18]^

## 5. Conclusion

DeepSeek is perceived by medical students as a promising and attractive tool with the potential to enhance academic and research productivity. However, concerns about reliability, ethics, and skill development must be addressed through education, transparency, and institutional support. In the future, longitudinal studies could employ network science or machine learning approaches to model the dynamic interactions between the use of AI tools and academic outcomes. Meanwhile, medical education should systematically integrate AI literacy, provide structured training on tools such as DeepSeek, and establish clear ethical guidelines to promote responsible AI use. By doing so, educators can help students harness the benefits of AI while mitigating its risks, ultimately fostering a more innovative and critical approach to research in the medical field.

## Acknowledgments

The authors would like to thank Xiang Wang, Zhehui Ma, Junjie Xie, and Yiran Li for their help in collecting the data. We also would like to thank all participants during the investigational period for their cooperation.

## Author contributions

**Conceptualization:** Jing Ni, Fan Yang, Lingqiong Jiang.

**Data curation:** Wenjie Xuan, Zhuoyi Wu, Lingqiong Jiang.

**Formal analysis:** Jing Ni, Fan Yang.

**Funding acquisition:** Jing Ni.

**Investigation:** Wenjie Xuan, Zhuoyi Wu.

**Methodology:** Jing Ni, Lingqiong Jiang.

**Supervision:** Jing Ni.

**Validation:** Jing Ni, Lingqiong Jiang.

**Writing – review & editing:** Jing Ni.

**Writing – original draft:** Lingqiong Jiang.
